# A survey on computational taste predictors

**DOI:** 10.1007/s00217-022-04044-5

**Published:** 2022-05-26

**Authors:** Marta Malavolta, Lorenzo Pallante, Bojan Mavkov, Filip Stojceski, Gianvito Grasso, Aigli Korfiati, Seferina Mavroudi, Athanasios Kalogeras, Christos Alexakos, Vanessa Martos, Daria Amoroso, Giacomo Di Benedetto, Dario Piga, Konstantinos Theofilatos, Marco Agostino Deriu

**Affiliations:** 1 PolitoBIOMedLab, Department of Mechanical and Aerospace Engineering, Politecnico di Torino, Turin, Italy; 2 Dalle Molle Institute for Artificial Intelligence (IDSIA-USI/SUPSI), Lugano-Viganello, Switzerland; 3 InSyBio PC, Patras, Greece; 4 Athena Research Center, Industrial Systems Institute, Patras, Greece; 5 Department of Plant Physiology, Institute of Biotechnology, University of Granada, Granada, Spain; 6 Enginlife Engineering Solutions, Turin, Italy; 7 7hc srl, Rome, Italy; 8 Faculty of Computer and Information Science, University of Ljubljana, Ljubljana, Slovenia; 9 GIPSA-lab, F-38000, Université Grenoble Alpes, Grenoble, France; 10 Department of Nursing, School of Rehabilitation Sciences, University of Patras, Patras, Greece

**Keywords:** Machine learning, Taste prediction, Molecular descriptors, Small compounds, Tastants, Food

## Abstract

Taste is a sensory modality crucial for nutrition and survival, since it allows the discrimination between healthy foods and toxic substances thanks to five tastes, i.e., sweet, bitter, umami, salty, and sour, associated with distinct nutritional or physiological needs. Today, taste prediction plays a key role in several fields, e.g., medical, industrial, or pharmaceutical, but the complexity of the taste perception process, its multidisciplinary nature, and the high number of potentially relevant players and features at the basis of the taste sensation make taste prediction a very complex task. In this context, the emerging capabilities of machine learning have provided fruitful insights in this field of research, allowing to consider and integrate a very large number of variables and identifying hidden correlations underlying the perception of a particular taste. This review aims at summarizing the latest advances in taste prediction, analyzing available food-related databases and taste prediction tools developed in recent years.

**Supplementary Information:**

The online version contains supplementary material available at 10.1007/s00217-022-04044-5.

## Introduction

Taste is a crucial sense involved in the perception of food and is a sensory modality that participates in the regulation of the intake of substances, avoiding indigestible or harmful ingredients and identifying safe and healthy nutrients. Taste is determined by the gustatory system and participates in the overall perception of the flavor together with smell (olfactory system) and touch (trigeminal system) [1]. Chemicals derived from food ingestion trigger the taste perception process, starting in the oral cavity, where they bind specific proteins placed on the taste buds of the tongue [2]. The five principal tastes are bitter, sweet, sour, umami, and salty, with each one being detected by specific receptors. Other tastes, such as fat taste, might be considered basic ones, since they arise from the combination of somatosensory and gustation perceptions [3, 4]. Each taste is linked to a vital somatic function. In general, the sweet taste is associated with the presence of energy-rich food; the bitter taste is usually linked to potentially dangerous compounds and unpleasant flavor; umami is connected with the protein content in food; sour helps in the detection of spoiled food and acid tastants in general; finally, salty taste monitors the intake of sodium and other minerals [5]. Moreover, the taste is also supported by the sense of smell in the evaluation of foods or substances, and chemosignal detection is used by animals and humans to identify threats [6, 7]. As an example, repulsive odors to humans, such as the ones generated from cadaverine, putrescine, and other biogenic diamines, indicate the presence of bacterial contamination [8]. Taste sensation relies on the affinity of taste compounds for taste receptors depending on their structure. Since small variations in tastant chemistry result in drastic modifications of perceived taste, ligand-based methods, merging molecular descriptors and taste information, represent powerful data-driven tools to effectively implement machine learning (ML) algorithms with the capacity to predict taste. Such methods can be applied, for example, to screen huge databases of small compounds (e.g., ZINC15, DrugBank, and ChEMBL) to select promising tastants or to rationally drive the design of novel compounds with specific functional properties and a desired taste.

Nutritious foods usually have an appetitive taste, e.g., sweet, umami, and lower concentrations of sodium and acids, whereas toxic substances generally present an unpleasant flavor, such as bitter tastants, high concentrations of sodium, and sour taste stimuli. Moreover, a healthy diet, such as the Mediterranean one, has been associated with beneficial impacts on human health status [9, 10]. Taste prediction is therefore of paramount importance not only for the food industry but also for the medicine, pharmaceutical and biotechnology sectors. Regarding the industrial food sector, sensory evaluation is commonly applied to access the flavor of foods. Usually, it involves the measurement and evaluation of the sensory properties of foods and other materials [11, 12]. The type of analysis role is crucial to address specific consumers' needs or market demands, evaluate food products, ensure high-quality products, and establish the minimum shelf life of a product, food obsolescence, or spoilage [13]. However, traditional methods cannot evaluate investigated food in a precise quantitative way, but only in a qualitative manner [14]. Moreover, the sensory evaluation typically requires many sensory professionals to reach a more objectiveness, with consequent problems generated by intra- and inter-operator variability, long lead times, and high costs [11, 14]. Thus, it is crucial to develop rational, fast, and cost-effective methods to assess the food quality and its related properties, including taste. Moreover, concerning the nutritional and health field, the sweetness prediction might point out novel promising sweeteners with low caloric value to reduce the caloric intake derived from the ingestion of naturally occurring or added sugars, in line with the recommendations of the World Health Organization [15]. Indeed, the excessive consumption of added sugars is normally linked to an increase in body weight [16], obesity [17, 18], and severe pathologies, such as diabetes or cardiovascular diseases [19, 20]. Other examples linked to the importance of taste prediction include bitter masking molecules. Indeed, the bitter taste is one of the main problems for pharmaceutical industries due to its unpleasant taste, which represents one of the main barriers to taking medications, especially for children and the elderly population [21]. Furthermore, a change in taste perception might be caused by the onset of other pathologies, such as in the case of the loss and/or impairment of taste function after COVID-19 infection [22].

This work aims to summarize the main recent efforts in the in-silico taste prediction, starting from an overview of the major taste or food-related molecules databases and the implemented ML-based prediction tools.

## Taste and food-related databases

The first essential step for the implementation of ML-based tools is the definition of reliable and as comprehensible as possible databases (DBs) with information concerning the taste of each entry. In the past years, several databases of small compounds related to their specific taste sensations in foods have been developed. In this section, the authors pinpoint the major databases and their characteristics, which are summarized in Table 1.

**Table 1:** Summary of the main taste databases with weblinks, present tastes, the relative number of molecules, and the possibility to download data

Reference	Link	Taste	No	Download
SuperSweet [23]	/	Sweet	8000	No
SweetenersDB [24]	https://bit.ly/32fG9af	Sweet	316	No
BitterDB [25]	https://bit.ly/3FinsB6	Bitter	1041	Yes
BTP640 [26]	https://bit.ly/3pogTrj	Bitter	320	Yes
		Non-Bitter	320	
Rodgers Database [27]	/	Bitter	682	/
Umami Database	https://bit.ly/3FhePa1	Umami	800	No
UMP442 [28]	https://bit.ly/3yK6EAk	Umami	104	Yes
		Non-Umami	304	
TastesDB [29]	/	Sweet	435	/
		Bitter	81	
		Tasteless	133	
Fenaroli’s Handbook of Flavor Ingredients [30]	/	Sweet	426	/
		Bitter	33	
		Tasteless	3	

In Table 2, other databases, which do not contain precise information regarding the taste associated with each element but are related to food ingredients and widely used in taste prediction, are reported.

**Table 2:** Summary of the main databases related to food or commonly used by taste prediction tools, with weblinks, the number of compounds collected in each DB, and the possibility to download data

Reference	Link	No	Download
FooDB	https://foodb.ca/	28 k	Yes
Super Natural II [31]	https://bioinf-applied.charite.de/supernatural_new/	326 k	No
FlavorDB [32]	https://cosylab.iiitd.edu.in/flavordb/	~ 26 k	No
PhytoHub	http://phytohub.eu/	1863	Yes
Phenol-Explorer [33]	http://phenol-explorer.eu/	501	Yes
BIOPEP-UWM [34]	https://biochemia.uwm.edu.pl/	4321	No
ChEMBL [35]	https://www.ebi.ac.uk/chembl/	~ 17 M	Yes
DrugBank [36]	https://go.drugbank.com/	~ 500 k	Yes
ZINC15 [37]	https://zinc.docking.org/	230 M	Yes
PhytoLab	https://www.phytolab.com/en/	~ 1300	Yes
Natural product atlas [38]	https://www.npatlas.org/	~ 24 k	Yes

Most used and tested databases (DBs) are described in detail in the following paragraphs.

### SuperSweet

SuperSweet (https://bioinformatics.charite.de/sweet/) contains more than 8000 artificial and natural sweet compounds [23]. The dataset includes the number of calories, the physicochemical properties, the glycemic index, the origin, the 3D design, and other information regarding molecular receptors and targets. Sweet-tasting chemicals were taken from the literature and freely accessible data sets. The web server interface offers a very user-friendly search and a sw*eet tree* which groups the sweet substances into three main families, (carbohydrates, peptides and small molecules).

### SweetenersDB

SweetenersDB (http://sebfiorucci.free.fr/SweetenersDB/) is a database of 316 sugars and sweeteners from 17 chemical families. Compounds were aggregated with their 2D structure or with a 3D structure using Marvin Sketch (ChemAxon) and the protonation state was defined at a pH of 6.5, according to the common pH value found in the saliva. Two natural compounds of the SweetenersDB are also present in Super-Natural II (entries: 105.620, 325.102) [24].

Each sweetener has also an assigned sweetness value, indicated as logS. This value is the logarithm of the ratio between the concentrations of the considered compound and sucrose, used as a reference. In this way, this value reflects the relative sweetness of a specific compound if compared to sucrose. From a physicochemical analysis of the database, an intense sweetener has low molecular weight and a hydrophobic core. Natural sweeteners are the molecules with the highest molecular weight, and they are capable of forming more hydrogen bonds than all the sweeteners in the SweetenersDB.

### BitterDB

BitterDB (http://bitterdb.agri.huji.ac.il/dbbitter.php) is a free source containing information about bitter taste molecules and their receptors [39]. In 2019, an upgrade of the database was made with an increase in the number of compounds (from the initial 550–1041) and the insertion of new features, including for example data belonging to different species rather than humans (mouse, cat, and chicken).

BitterDB contains now about 1041 molecules collected from over 100 publications. For each compound, the DB provides different information, such as molecular properties, identifiers (SMILES, IUPAC name, InChIKey, CAS number, and the primary sequence of proteins), cross-links, qualitative bitterness category (i.e., bittersweet, extremely bitter, slightly bitter, etc.), origin (from a natural source or synthetic), and different file formats for download (SDF, image, smiles, etc.). Furthermore, toxicity data were added from the Acute Oral Toxicity Database when available, reporting experimental rat LD_50_ values as described in the previous literature [40].

Most of the SMILES were taken from PubChem and the remaining ones were generated through the CycloPs server, after drawing the molecules on ChemSketch or ChemAxon. Regarding the other identifiers, the ones not available in PubChem were processed using RDKit (http://www.rdkit.org).

### BTP640

BTP640 collects 320 experimentally confirmed bitter peptides and 320 non-bitter peptides. Bitter peptides were retrieved from various literature and peptides including ambiguous residues (e.g., B, X, Z, and U) or duplicated peptide sequences were discarded. Since few experimental data concerning non-bitter peptides are available, the negative dataset was built starting from BIOPEP dataset, which contains biologically active peptide sequences (4304) widely used in the food and nutrition field (Minkiewicz et al., 2019). From this dataset, 320 peptides were randomly extracted to build the negative dataset.

### Rodgers database

This database was collected from previous literature and patents, including studies in BIOSIS, Food Science and Technology Abstracts, databases of internal reports at Unilever and Derwent World Patents Index (WPIDS) [27]. Structures were obtained from SciFinder, where possible, or constructed with ChemDraw. After the removal of synthetic analogs, the final database contains 649 bitter molecules. It is worth mentioning that additional 33 molecules were then considered by the authors and made public in the original paper, whereas the other 649 remain non-public. Unfortunately, no webserver or online data repository is available for this database.

### Umami Database

The Umami Database (https://www.umamiinfo.com/umamidb/) is developed by the Umami Information Center, founded with the support of the Umami Manufacturers Association of Japan in 1982. The Umami Database was created with the idea of providing information about the umami taste in foods and, currently, about 800 items are listed in the database. Amino acids in foods are mainly of two types, i.e., ones joined together to form proteins and free amino acids, that have a more pronounced flavor. In this context, free glutamate has a remarkable umami taste. Umami Database reports also the score of free glutamate and other free amino acids which affect food taste. In addition, there are inosinate and guanylate scores, which synergistically increase umami perception. Sources for the Umami Database include public academic papers and scores analyzed by a research laboratory upon request of the Umami Information Center.

### UMP442

This dataset was constructed for the development of the iUmami prediction tool [28]. The umami set merges several experimentally validated umami peptides from the literature [41–46] and the BIOPEP-UWM database [34]. On the other hand, the non-umami peptides dataset is made by the bitter peptides from the positive set of BTP640 [47]. After removing peptides with non-standard letters and redundant sequences, the final UMP442 database collects 140 umami and 304 non-umami peptides. The dataset was made publicly available on GitHub (https://github.com/Shoombuatong/Dataset-Code/tree/master/iUmami).

### TastesDB

TastesDB is an experimental database comprising 727 chemicals with their respective experimental taste class, retrieved from several scientific publications [29]. Since all incorrect molecules or those with problematic molecular structures were removed, the final TastesDB contains 649 molecules, specifically 435 sweet, 81 bitter, and 133 tasteless (see also Table S1 of the original paper for further details). For each entry of the database, the DB provides the commercial name, the SMILE, the tasting class (sweet, tasteless, and bitter), and the literature reference.

## Taste prediction with machine learning

In the past years, several studies have developed ML-based algorithms to predict the taste of specific molecules starting from their chemical structure. In this section, we will review in detail the main recent literature in the field of taste prediction. Where no precise name has been defined for the tools discussed, we have decided to use the first author name of the reference publication for simplicity (Table 3).

**Table 3:** Summary of the main recent taste prediction tools, including methods, datasets, and molecular descriptors employed (see also Table S1 for further information)

Reference	Method	Taste	No	Descriptors
Chéron Sweet Regressor [24]	Sweet Regressor (RF, SVR)	Sweet	316	Dragon
Rojas Sweet Predictor [29]	Sweet Classifier (QSTR)	Sweet	435	ECFP, Dragon
		Non-Sweet	214	
Goel Sweet Regressor [48]	Sweet Regressor (GFA, ANN)	Sweet	487	Material Studio
e-Sweet [49] [https://bit.ly/3wFy4ER]	Sweet Classifier (KNN, SVM, GBM, RF, DNN)	Sweet	530	ECFP
		Non-Sweet	850	
Predisweet [50] [https://bit.ly/3reop7a]	Sweet Regressor (AB)	Sweet	316	Dragon, RDKit, Mordred, ChemoPy
BitterX [51] [https://bit.ly/3wJYa9O]	Bitter Classifier (SVM)	Bitter	539	[52]
		Non-Bitter	539	
BitterPredict [53] [https://bit.ly/3igrzmQ]	Bitter Classifier (AB)	Bitter	691	Canvas (Schrödinger)
		Non-Bitter	1952	
e-Bitter [54] [https://bit.ly/3epWzQq]	Bitter Classifier (KNN, SVM, RF, GBM, DNN)	Bitter	707	ECFP
		Non-Bitter	592	
iBitter-SCM [26] [https://bit.ly/2VGyXAg]	Bitter Peptides Classifier (SCM)	Bitter	320	Dipeptide composition (DPC)
		Non-Bitter	320	
BERT4Bitter [55] [https://bit.ly/2WecTxf]	Bitter Peptides Classifier (BERT)	Bitter	320	Dipeptide composition (DPC)
		Non-Bitter	320	
iBitter-Fuse [56] [https://bit.ly/3BmC547]	Bitter Peptides Classifier (SVM)	Bitter	320	DPC, AAC, PAAC, APAAC, AAI
		Non-Bitter	320	
BitterIntense [57]	Bitter Intensity Classifier (XGBoost)	VB	246	Canvas (Schrödinger)
		NVB	404	
iUmami-SCM [28] [https://bit.ly/3hJs9uf]	Umami Classifier (SCM)	Umami	140	Dipeptide composition (DPC)
		Non-Umami	304	
BitterSweetForest [58]	Bitter/Sweet Classifier (RF)	Sweet	517	RDKit (Binary fingerprints)
		Bitter	685	
BitterSweet [59] [https://bit.ly/3rd7Att]	Bitter/Sweet Classifier (AB, RF)	Bitter	918	Canvas, Dragon, ECFP, ChemoPy
		Non-Bitter	1510	
		Sweet	1205	
		Non-Sweet	1171	
		Non-Umami	304	
VirtualTaste [60] [https://bit.ly/2UfVFPi]	Multi-taste classifier (RF)	Sweet	2011	*Not reported*
		Bitter	1612	
		Sour	1347	

In the following, each of the aforementioned tools is examined in detail, dividing the discussion into a brief introduction, the “*Data preparation and model construction*” section and the “*Model performance*” section.

### Sweet prediction

#### Chéron sweet regressor

In this work, a Sweet Predictor was created using a new QSAR model [24]. This model, also applied to external datasets (SuperSweet and SuperNatural II), allowed to point out the main physio-chemical features of sweeteners related to their potency.

##### Data preparation and model construction

The curated dataset of sweet compounds resulted in the creation of the SweetenersDB, which is constituted of 316 compounds with known sweetness values relative to sucrose (see *Taste and Food-Related Databases* chapter for further details). The compounds' SMILES were first collected in a 2D database, and subsequently, 3D representations were created using Marvin, ChemAxon (https://www.chemaxon.com), choosing the three with the lowest energy. The protonation state was set at the physiological salivary pH value (6.5).

Dragon descriptors (http://www.talete.mi.it/products/dragon_description.htm) were calculated for both the 2D and 3D databases. All features with a correlation greater than 0.9 were removed, obtaining 244 descriptors for the 2D molecules and 265 descriptors for the 3D structures. Finally, all descriptors were normalized.

The dataset was randomly divided with a 70:30 ratio and the leave-one-out method was used for the cross-validation. Support Vector Regression (SVR) and Random Forest (RF) were optimized on the training set, and the test set was used for the model performance evaluation.

##### Model performance

Performance evaluation was obtained using the squared of the correlation coefficient (R^2^). Notably, the SVR reached a slightly better performance than RF on the test set. It is worth mentioning that the models on 2D and 3D datasets reached similar performances, suggesting the 2D approach as the best option for fast screening, since it is much less time-consuming. More in detail, the RF 2D, SVR 2D, RF 3D, and SVR 3D models obtained correlation coefficients on test sets of 0.74, 0.83, 0.76, and 0.85, respectively.

To evaluate the model applicability domain, SweetenersDB was compared with SuperSweet and SuperNatural II. Interestingly, 99.5% of the molecules from SuperSweet are similar to structures in SweetenersDB, whereas only about 34% of the SuperNatural II database belong to the chemical space defined by SweetenersDB. This analysis confirmed the importance of associating an applicability domain to a prediction model to measure the reliability of the prediction.

#### Rojas sweet predictor

The present Quantitative Structure-Taste Relationship (QSTR) model is a specialist framework created to foresee the pleasantness of synthetic compounds [29]. It can likewise be utilized to gain a comprehensive understanding between atomic design and pleasantness and defining novel sugars. This sweetness prediction model is the first QSTR model that considers both molecular descriptors and extended connectivity footprints, performing a structure similarity analysis in combination with the model prediction.

##### Data preparation and model construction

The starting dataset is TastesDB (see also *Taste and Food-Related Databases* chapter for further details). The dataset includes 649 molecules: 435 sweet, 81 bitter, and 133 tasteless; the latter two classes were combined into a non-sweet class. Extended-connectivity fingerprints (ECFPs) [61] and classical molecular descriptors, i.e., Dragon 7 (3763 total descriptors) (https://chm.kode-solutions.net/pf/dragon-7-0/), were used to describe the molecules of the dataset. In all cases, the 2D representation was preferred to the 3D one, to get a conformation-independent molecular representation.

Exploration of the data and similarity analysis was performed using the Multidimensional Scaling (MDS), whereas the Partial Least Squares Discriminant Analysis (PLSDA) and N-Nearest Neighbors (N3) were employed as classifiers. Finally, the V-WSP unsupervised variable reduction method and the Genetic Algorithms-Variable Subset Selection (GA-VSS) technique were used as dimensionality reduction techniques to retrieve the most informative molecular descriptors.

The dataset was divided into three parts, maintaining the proportion of the classes: the training set consisting of 488 compounds (161 non-sweet and 327 sweet molecules), the test set consisting of 161 molecules (53 non-sweet and 108 sweet molecules), and finally, the last part was used as an external dataset. Moreover, a fivefold CV was employed for the GA-VSS and the Monte Carlo (leave-many-out) random sub-sampling validation of the system. These methods iteratively and randomly divided the molecules into training (80%) and evaluation (20%) sets.

##### Model performance

Specificity (SP), sensitivity (SN), and non-error rate (NER), which is more efficient in the case of unbalanced datasets, were used as performance metrics. The two final models, made with six molecular descriptors, were chosen based on the NER classification parameter. Since PLSDA and N3 are based on distinct methods and descriptors, a consensus analysis was employed to improve prediction [62]. Therefore, a molecule was classified if both models showed the same result and not classified otherwise.

Performance in calibration (SE = 79.2%, NER = 85.2%, SP = 91.3%, not assigned = 33%), in cross-validation (SE = 77.2%, NER = 83.1%, SP = 89.0%, not assigned = 32%), in the Monte Carlo validation (NER = 88.7%, SE = 92.7, SP = 84.8%, non-assigned = 20.5%), and in the 161 test molecules (NER = 84.8%, SE = 88.0%, SP = 81.6%, non-assigned = 19.3%) confirm the model stability. The consensus analysis improved the overall performance of the model. Notably, the model calibration was performed only on a cluster of the complete dataset, that was derived from the MDS analysis. The remaining part of the original dataset was classified using similarities scores combined with the aforementioned models.

#### Goel sweet regressor

The present Sweet Regressor tool is a QSAR model able to estimate the relative sweetness level of a test compound with respect to the sweetness of sucrose [48]. This tool can act as a pre-processing step in the design of new sweeteners by pointing out their crucial structural requirements.

##### Data preparation and model construction

The dataset was collected from several publications [63–67], resulting in 487 unique molecules with relative sweetness compared to sucrose (ranging from − 0.699 to 5.334) calculated as described for the SweetenersDB (see also the *Taste and Food-Related Databases* chapter). Compounds SMILE were converted to 3D structures and Material Studio v6.0 was used to calculate 564 molecular descriptors. After performing a correlation analysis, only 61 descriptors were maintained and, after removing outliers, the remaining 455 molecules were randomly divided between the training and test sets (70:30 ratio).

Two QSAR models were developed using Artificial Neural Network (ANN) and Genetic Function Approximation (GFA) algorithms.

##### Model performance

Performance was assessed using the correlation coefficient for the training set, leave-one-out method and test set (*R*_training_^2^, *R*_cv_^2^ and *R*_test_^2^), Mean Absolute Error (MAE), Mean Absolute Percentage Error (MAPE), and Mean Square Error (MSE). Statistical parameters of ANN (*R*_training_^2^ = 0.889, *R*_test_^2^ = 0.831) and GFA with linear spline (*R*_training_^2^ = 0.864, *R*_test_^2^ = 0.832) were comparable, and both QSAR models demonstrated a reasonable prediction accuracy. GFA allows the development of numerous models, autonomously selecting features and broadening the number of terms used in model construction and easily interpreting the data. On the other hand, ANN can further explain hidden relationships between complicated data and depict patterns and trends, but it lacks interpretability.

#### e-Sweet

e-Sweet is a free tool to predict the sweet taste of analyzed chemicals and their relative pleasantness (RS) [49].

##### Data preparation and model construction

The entire dataset includes 1380 compounds, divided into sweet and non-sweet. Sweet compounds are 530 sugars curated from SuperSweet, SweetenersDB and previous literature [29, 58], while 850 non-sweet comprise 718 entries from BitterDB and 132 recovered from the literature [29].

The 80:20 data splitting scheme was adopted, resulting in 883 compounds for training and internal fivefold CV and 221 compounds for the test. Features were built using Extended-Connectivity Fingerprints (ECFP) [61] and subsequently selected by their importance in a trained RF model. Implemented algorithms comprised KNN, SVM, GBM, RF, and DNN with different splitting procedures (19 different splits for the former four models and 3 different splits for DNN) to reduce the bias yielded by specific splits. A total of 1312 models were first assessed individually and subsequently combined to form a pool of 4 Consensus Models (CM), that leverage the combination of individual models to improve overall classification performance.

##### Model performance

Model performance was assessed based on widely used metrics [F1-score, specificity, sensitivity, accuracy, precision, Matthews Correlation coefficient (MCC), and Non-Error Rate (NER)]. F1-score metric was chosen as the final algorithm selection criterion and, subsequently, a Y-randomization test was performed for a direct assessment of model robustness.

The split-averaged performance of the best CM on the test set reached 91% of accuracy, 90% precision, 94% specificity, 86% sensitivity, F1-score of 88%, MCC of 81%, and 90% NER, all with 95% confidence intervals of ± 1%.

#### Predisweet

Predisweet is a free-available web server (http://chemosimserver.unice.fr/predisweet/) capable of predicting sweet taste and the relative sweetness (in logarithmic scale) of compounds [50]. The applicability, reliability, and decidability domains have been used to estimate the quality of each prediction.

##### Data preparation and model construction

The tool is based on the SweetenersDB, collecting 316 compounds with their relative sweetness (see also *Taste and Food-Related Databases* chapter) [24]. Two other databases, namely Super-Natural II database [31] and the *phyproof* catalog from PhytoLab (https://www.phytolab.com/en/), were considered as an external dataset (4796 natural compounds).

Each compound was collected as SMILE and sanitized using RDKit (https://www.rdkit.org/). The protonation state was predicted using ChemAxon (http://www.chemaxon.com/) at the physiological pH of saliva (pH = 6.5). Molecules were standardized using the *flatkinson* standardiser (https://github.com/flatkinson/standardiser) and further processed with a Python package, removing salts and applying specific rules to normalize the structures. Molecular descriptors were calculated using Dragon v6.0.38, RDKit, Mordred [68] and ChemoPy [69] packages. Descriptors from the latter three methods (506) were defined as “open source” descriptors, as opposed to Dragon ones (635).

The Sphere Exclusion clustering algorithm divided the SweetenersDB into the training set (252 compounds) and the test set (64 compounds).

Several regression algorithms were tested, including Support Vector Machine (SVM), k-Nearest Neighbors (KNN), Random Forest (RF), and Adaptative Boosting with a Decision Tree base estimator (AB), and the fivefold CV was employed to avoid asymmetric sampling and overfitting.

##### Model performance

Predictive performance is assessed through Golbraikh and Tropsha criteria [70], and the AdaBoost Tree was considered the best method for both models. The obtained models reach R^2^ higher than 0.6 (0.74 for the Open-source and 0.75 for the Dragon model) and Q^2^ higher than 0.5 (0.84 for the Open-source and 0.79 for the Dragon model) for both models (detailed performance reported in Table 1 and Table S3 of the original paper). Notably, since less information was available regarding potent sweeteners, developed models perform worst for high sweetness values. The open-source and the Dragon models reached similar performances showing good prediction on the test set. Therefore, the open-source version was used for the webserver (http://chemosimserver.unice.fr/predisweet/) implementation of the algorithm.

The quality of the prediction for each query is evaluated based on three metrics: (i) the *applicability domain*, which measures if the investigated compound is in the range of descriptors of the training set, (ii) the *reliability domain*, which considers the density of information around the compound, and (iii) the *decidability domain*, which is the confidence of the prediction. The resulting quality of the prediction is also reported for the user when using the webserver platform.

### Bitter prediction

#### BitterX

BitterX is a web-based platform (http://mdl.shsmu.edu.cn/BitterX/) available for free [51]. This tool implements two different models, i.e., the *bitterant verification model*, which allows the identification of a bitter compound, and the *TAS2R recognition model*, which predicts the possible human bitter taste receptors, among the 25 known TAS2Rs. Such predictions were validated experimentally.

##### Data preparation and model construction

The interactions between the TAS2Rs and bitter compounds were curated from PubMed and BitterDB. A total of 539 bitter compounds were obtained to constitute the positive set, and their molecular structures were achieved from Pubchem. The negative set included 20 true non-bitterants (in-house experimental validation) and 519 molecules from the Available Chemicals Directory (ACD, http://www.accelrys.com). The final dataset contained 1078 compounds, equally divided into positive and negative bitter molecules. Molecular structures were obtained from PubChem and processed with in-hose program Checker and ChemAxon’s Standardizer (http://www.chemaxon.com). On the other hand, the initial dataset for the TAS2R prediction model was taken from the literature and includes 2379 negative and 260 positive bitterant–TAS2R interactions. Due to the huge difference between the two dataset sizes, all the 260 non-redundant experimentally verified bitterant–TAS2R interactions were considered, while just 260 bitterant–TAS2R couples were selected as negatives to balance the dataset.

Physiochemical descriptors for compounds were chosen based on the Handbook of Molecular Descriptors [52], resulting in 46 and 20 descriptors for the bitterant verification and TAS2R recognition models, respectively. Moreover, 15 descriptors were used for the TAS2R representation in the TAS2R recognition model. The descriptors were chosen using a Feature Selection (FS) method based on a Genetic Algorithm (GA).

BitterX employs the support vector machine (SVM) classifier: the training was divided into two categories (+ 1 and − 1) that represent the classification between bitter and non-bitter (and the bitterant–TAS2R interaction or not). The SVM + sigmoid method was implemented to value the probability that a molecule is bitter in the bitterant authentication and the probability of a bitterant binding to TAS2Rs in the TAS2R recognition model.

Data for algorithm training and test were used by adopting an 80:20 splitting strategy. For the bitterant verification model, this results in 862 and 216 compounds for training and test, respectively. To avoid any error derived from a particular data splitting, the other two partitions were made randomly from the general database, always following the equipartition between bitter and non-bitter molecules. Similarly, the TAS2R recognition dataset was also divided with a 4:1 ratio. Finally, a fivefold CV ensured the robustness of the classification algorithm.

##### Model performance

To evaluate and compare the model, four indices were calculated: precision, accuracy, sensitivity, and specificity. Furthermore, the trade-offs between SE and SP were assessed by performing the ROC curve and calculating the AUC value.

Considering both the training (5-CV) and test sets, the *bitterant verification model* reached specificity, precision, and sensitivity values above 90%, AUC above 94%, and accuracy above 87%, whereas the *TAS2R recognition model* reached above 76% for the accuracy, above 75% for precision and specificity, above 78% for sensitivity, and above 81% for AUC.

#### BitterPredict

BitterPredict is a bitter prediction tool published in 2017 [53]. This work is developed in the commercial MATLAB environment and the code is available on GitHub (https://github.com/Niv-Lab/BitterPredict1). The users should provide an Excel or CSV file with calculated properties by the commercial Schrödinger software and QikProp package.

Among random molecules screened by the tool, a high percentage is represented by bitter compounds. These include many synthetic molecules (66% of drugs are bitter) and natural compounds (up to 77%). It is worth mentioning the relatively high percentage of bitter food (38%), considering the natural aversion of humans towards the bitter taste.

Despite its great functionality, high accuracy (~ 80%), and the ability to screen a general chemical space, this tool presents some limitations, including (i) the prediction of only molecules in defined chemical space, named Bitter Domain, (ii) the inability to discriminate between weak and strong bitter compounds, and (iii) an unbalanced dataset (positive set three times smaller than the negative set).

##### Data preparation and model construction

The dataset was processed using Maestro, Epik, and LigPrep (Schrödinger), removing uninterested structures and assigning the correct protonation state according to pH 7.0 ± 0.5. Then, non-neutralized molecules and molecules with identical descriptors were removed from the dataset to allow the calculation of the QikProp descriptors and obtain a non-redundant dataset. The whole prediction was made within a restricted chemical space called *Bitter Domain* to identify a region in which 97% of the bitter molecules is included. This domain is defined by a hydrophobicity (AlogP) range of − 3 ≤ AlogP ≤ 7 and a molecular weight MW ≤ 700. This preparation procedure was applied to each database considered to build the final dataset.

Bitter Set (*positive set*) includes all molecules considered as bitter (691): 632 structures from BitterDB and 59 molecules from literature [71]. On the other hand, the Non-Bitter Flavors set (*negative set*) consisting of 1917 non-bitter molecules: “probably not bitter” compounds gathered from Fenaroli’s handbook of Flavor ingredients (1451), sweet (336), and tasteless (130) molecules from Rojas et al. The *validation set* consisted of Bitter New, i.e., 23 molecules stored recently from several publications to increase BitterDB, UNIMI set, i.e., 56 synthesized molecules, and the Phytochemical Dictionary, consisting of 26 non-bitter and 49 bitter compounds inside the Bitter Domain. Moreover, a set of data was collected for the *sensory evaluation*. 1047 molecules were retrieved from the Sigma-Aldrich flavors and fragrances catalog (https://www.sigmaaldrich.com/IT/it/applications/food-and-beverage-testing-and-manufacturing/flavor-and-fragrance-formulation). After data curation, 264 entries were selected as the Bitter Domain. Finally, a data set for *prospective prediction* was collected merging DrugBank approved drugs, FooDB, Natural Products Dataset from ZINC15 and ChEBI. Compounds in the Bitter Domain from these widely used DBs were 1375, 13,588, 27,474, and 27,015, respectively.

Molecular descriptors (59) were calculated with Canvas (Schrödinger) and QikProp (Schrödinger).

The final input dataset was divided into 30% (test set) and 70% (training set) randomly, following the hold-out method and preserving the original proportions. To avoid overfitting, models were optimized by evaluating their performance only in the hold-out test.

The algorithm implemented by BitterPredict is AdaBoost. The ensemble method models used are Fitensemble and TreeBagger, which combines the outcomes of several decision trees, decreasing the impacts of overfitting and enhancing the generalization ability of the model.

##### Model performance

The two parameters applied to evaluate the classification models were sensitivity (SE) and specificity (SP). For the training set, the SE was 91% and SP 94%, for the test set SE was 77% and SP 86%. Furthermore, a model evaluation based only on the non-bitter datasets was made among sweet, tasteless, and non-bitter flavors. In this context, the dataset that shows a better specificity was the non-bitter flavors, with a value of 86%.

The BitterPredict study also analyzed the impact of diverse descriptors estimating their contribution in reducing the error. Notably, the most important descriptor was the total charge and most of the bitter molecules were positively charged presenting an ammonium ion at physiological pH. Moreover, QikProp descriptors linked to the compound toxicity seem to have a greater impact on the model if compared to general properties descriptors.

The validation of BitterPredict was performed in three phases, as follows.

i. *Validation using external sets* (see *Data preparation and model construction* for further details) tested the algorithm on datasets never seen before by the algorithm to avoid overfitting. Excellent performance was achieved with a specificity of 69–85% and sensitivity of 74–98%.
ii. *Validation by literature mining* consisted of a selection of 60 compounds from a DrugBank set of FDA approved drugs, half of which with the best and half of which with the worst score of bitter prediction according to BitterPredict. The results from literature research indicated that almost 60% of the top 30 bitter molecules were declared to have a bitter taste, while only 20% of the 30 non-bitter molecules had a probable indication of a bitter taste.
iii. For the *validation by taste tests* (*sensory evaluation*), 12 participants were selected to evaluate the taste of 6 compounds predicted as non-bitter by BitterPredict among the 264 compounds taken from fragrances catalog and Sigma-Aldrich flavors (see also *Data preparation and model construction* section). None of the six compounds differed in bitterness from the control (water) with the Dunnett test (alpha = 0.05), whereas the Quinine (established bitter molecule) demonstrate a considerably higher bitterness compared to water.

The three validation protocols indicated that BitterPredict allows obtaining reliable and satisfactory performance both for the bitter and non-bitter prediction.

Finally, the BitterPredict classifier was applied to DrugBank approved, FooDB, and Natural Products Dataset from ZINC15 and ChEBI datasets (see also see *Data preparation and model construction*). The results highlight that the percentages of bitter molecules are, respectively, 65.94%, 38.36%, 77.21%, and 43.71%.

#### e-Bitter

Developed by the same research group that created e-Sweet [49], e-Bitter is a free graphic program published in 2018 for bitter prediction, which natively implements the ECFP fingerprint and the analysis of the structural features [54]. Differently from other works [51, 53], e-Bitter considers only experimentally confirmed non-bitterants, i.e., 592 compounds comprising tasteless, sweet, and non-bitter chemicals. e-Bitter code is publicly available (https://www.dropbox.com/sh/3sebvza3qzmazda/AADgpCRXJtHAJzS8DK_P-q0ka?dl=0).

##### Data preparation and model construction

The dataset contains experimentally confirmed 707 bitter compounds, derived mostly from BitterDB [25] and literature research [27, 29], and 592 non-bitter compounds (132 tasteless, 17 non-bitter, and 443 sweet). Sweet compounds were obtained from SweetnersDB, SuperSweet and previous literature [71, 72]. The same compounds but with a different taste or from different datasets, or compounds such as salts or ions, were excluded, while all structures containing common elements were retained. e-Bitter uses Extended-Connectivity Fingerprints (ECFPs) as molecular descriptors [61]. Similarly to e-Sweet [49], the implemented algorithms were KNN, SVM, GBM, RF, and DNN. Models were tested both with and without feature selection [73].

The splitting of the dataset follows the same criterion as previously described in e-Sweet: 1030 compounds (556 bitter and 474 non-bitter), i.e., 80% of the initial dataset, were employed as training data and for internal validation, while the remaining ones, consisting of 259 compounds (141 bitter and 118 non-bitter) were used for performance testing.

##### Model performance

The metrics employed to assess the model performance include precision, Matthews correlation coefficient (MCC), sensitivity, accuracy, specificity, F1-score, and ΔF1-score (difference between the F1-score in cross-validation and the test set). Moreover, the reliability of the developed models was accessed using the *Y*-randomization test, as for the e-Sweet model. Finally, an applicability domain based on the Tanimoto similarity was implemented to avoid non-reliable predictions on compounds highly diverse from compounds in the dataset.

Starting from an initial set of 1312 models, 96 averaged models (over adopted data partitioning schemes) and 9 consensus models were tested. Best performance was obtained by top average models trained with DNN3 (ACC = 92.0%, SP = 80.8%, SE = 98%, MCC = 82.3%, and F1-score = 94.1%).

#### iBitter-SCM

The iBitter-SCM tool is the first computational model that provides a prediction of the peptides’ bitter taste starting with their AA sequence independently from structural and functional information [26]. iBitter-SCM is freely available as a web server (http://camt.pythonanywhere.com/iBitter-SCM), and all codes and datasets are also on GitHub (https://github.com/Shoombuatong2527/Benchmark-datasets).

##### Data preparation and model construction

The dataset BTP640 (see also *Taste and Food-Related Databases* chapter for further information) includes 640 molecules equally divided between bitter and non-bitter (training set 80% and test set 20%).

Features representation was realized using the dipeptide composition (DPC). iBitter-SCM is based on the scoring card method (SCM), which enabled robust protein and peptide function prediction and analysis without any information regarding their structure and relying instead on the so-called propensity scores of individual peptides and amino acids. More in detail, after preparing a training dataset and an independent dataset, the workflow started by determining the initial propensity scores (init-DPS) of dipeptides using statistics and subsequently applying Genetic Algorithms (GAs) to refine and optimize the score to the so-called optimized dipeptide propensity score (opti-DPS). Finally, the individual amino acid propensity score was again extracted by statistical methods enabling the final discrimination between bitter and non-bitter peptides employing a weighted sum with opti-DPS. In a nutshell, these scores represent the link between peptide composition and function, by directly quantifying the contribution of individual amino acids on the physical–chemical characteristics. Furthermore, informative physicochemical properties (PCPs) of individual amino acids, i.e., their direct involvement in fundamental biological reactions and pathways, were taken from the amino acid index database (AAindex) [74].

##### Model performance

The model was assessed with several performance metrics: accuracy (ACC), specificity (SP), sensitivity (SE), and Matthew coefficient correlation (MCC) and AUC.

The performances of the opti-DPS and the init-DPS were compared using the tenfold cross-validation and the independent test. The best model (opti-DPS) was chosen based on the best performance on the tenfold CV and independent test sets (ACC = 84.38%, SE = 84.38, SP = 84.38, MCC = 68.8%, AUC = 90.4%). Notably, the best opti-DPS outperforms init-DPS with enhancements on ACC, SN, SP, and MCC, and iBitter-SCM, compared with other traditional ML models (KNN, NB, DT, SVM, and RF), demonstrated better performance and greater robustness.

#### BERT4Bitter

After creating iBitter-SCM, the same research group developed BERT4Bitter, a similar tool for the classification of bitter peptides [55]. BERT4Bitter dataset is publicly available and the developed model is freely accessible through a user-friendly web server interface (http://pmlab.pythonanywhere.com/BERT4Bitter).

##### Data preparation and model construction

The dataset used to develop the BERT4Bitter model is the same used for the iBitter-SCM method, i.e., the BTP640 [26] (see also *Taste and food-related databases* chapter for further details). Using the same 80:20 splitting ratio, the BTP640 dataset was randomly divided for training and testing.

The peptide sequence featurization was achieved through the natural language processing (NLP) techniques, specifically using Pep2Vec [75] and FastText [76]. Each of the 20 amino acids is considered as a word and each peptide sequence was translated into a sentence (an n-dimensional word vector). In the same framework, the importance of each amino acid in the analyzed sequences was evaluated with the TFIDF method [77].

Three different deep-learning-based models, i.e., convolutional neural network (CNN), long short-term memory (LSTM) neural network, and BERT-based model, were implemented using different numbers of layers (6, 5, and 12, respectively) and rationally compared.

##### Model performance

Model evaluation was accessed both in the tenfold CV and independent test set. According to the cross-validation performance, the BERT model outperformed the CNN and LSTM ones in all the evaluation metrics (ACC = 0.86, AUC = 0.92, SP = 0.85, SE = 0.868, and MCC = 0.72).

Notably, considering the independent test-set performance, BERT4Bitter outperformed the iBitter-SCM tool with ACC and MCC of 0.92 and 0.84, respectively, demonstrating a stronger predictive ability in discriminating bitter and non-bitter peptides.

#### iBitter-Fuse

After the development of iBitter-SCM and BERT4Bitter, the same research group implemented an improved bitter/non-bitter peptides predictor, called iBitter-Fuse [56]. This model overcomes some of the main drawbacks of the previous ones, including the generalization capability linked to the feature representation, overfitting, redundancy, and the overall performance. Exploiting several feature encoding schemes and customized algorithms to identify the most informative features, iBitter-Fuse outperformed both iBitter-SCM and BERT4Bitter, establishing itself, at the moment, as the best tool for the prediction of bitter peptides.

##### Data preparation and model construction

BTP640 was again used as starting dataset as done for iBitter-SCM and BERT4Bitter, and the same 80:20 partition scheme was applied to effectively compare their performance.

Five feature encoding methods, including dipeptide composition (DPC), pseudo amino acid composition (PAAC), amino acid composition (AAC), physicochemical properties from AAindex (AAI) and amphiphilic pseudo amino acid composition (APAAC), and a merged feature (DPC + PAAC + AAC + AAI + APAAC), were calculated to consider both composition and physicochemical properties. The model is based on an SVM algorithm and the feature selection was performed using a customized GA algorithm using self-assessment-report (GA-SAR) [78].

##### Model performance

The fused feature allows obtaining the best performance (ACC, MCC, and AUC) on the cross-validation, outperforming the other five feature encoding methods. To reduce the number of fused features (994), the GA-SAR was applied and 36 features were consequently maintained.

Performance evaluation demonstrated that iBitter-Fuse outperformed the previous tools for predicting the bitterness of peptides, i.e., iBitter-SCM and BERT4Bitter, suggesting that it is a more reliable and accurate tool. More in detail, the present SVM-based model reached ACC, SE, SP, MCC, and AUC of 93.0%, 93.8%, 92.2%, 85.9%, and 93.3%, respectively.

#### BitterIntense

BitterIntense is a unique tool able to quantify the bitter intensity of a query molecule, discriminating between two classes, i.e., “not very bitter” (NVB) and “very bitter” (VB) [57]. This tool is paramount not only for food research but also for pharma and biotechnology industries: the ability to predict the level of bitterness during the drug discovery process represents a promising opportunity for reducing delays, animal use, and financial costs. In fact, the intensely bitter taste is often associated with difficulties in taking medication, especially for children and elderly people. BitterIntense, published at the end of 2020, was also applied to widely known databases, such as DrugBank, and specific COVID-19 drug candidate datasets, highlighting interesting considerations regarding bitter intensity and toxicity.

##### Data preparation and model construction

The screening of bitter compounds was performed employing the rat brief-access taste aversion (BATA) model, obtaining 34 compounds. The dataset collects BitterDB and AnalytiCon’s repository of natural compounds and counts about 180 molecules with a specified bitter intensity. The bitter recognition threshold is 0.1 mM: below this concentration, the molecules were considered “very bitter” (VB), whereas above this value “not very bitter” (NVB). Molecules without quantitative information were assigned to VB/NVB classes according to the taste descriptions. A non-bitter database of 152 randomly selected compounds from the negative set of BitterPredict was added to NVB class.

Moreover, external datasets have been screened using the optimized model. Toxicity data include the FocTox dataset, i.e., extremely hazardous compounds and FAO/WHO food contaminants, and the CombiTox dataset, i.e., a combination of DSSTox-the Distributed Structure-Searchable Toxicity Database and Toxin and Toxin-Target Database version 2.0 (T3DB) [40]. Hepatotoxicity data were retrieved from FDA’s DILIrank dataset [79]. Finally, external datasets include DrugBank [36], consisting of approved and experimental drugs, COVID-19 drugs, and their targets retrieved from “Coronavirus Information. IUPHAR/BPS Guide to Pharmacology” and Natural products atlas (NPatlas, version 2019_08) [38].

SMILES were processed using Maestro (Schrödinger) (3D reconstruction, protonation at pH 7.0 ± 0.5, removal of additional molecules, and generation of conformers). Molecular descriptors were calculated using Canvas (Schrödinger) and were divided into three groups, i.e., Physicochemical, Ligfilter, and QikProp. Compounds not having one of these were excluded. Feature selection was performed using the feature importance gain score, obtaining a total of 55 features (from the starting 235).

The dataset was randomly divided into a training set (169 VB and 324 NVB), a test dataset (43 VB and 80 NVB), and the hold-out set for an external evaluation (31 VB and 74 NVB).

The algorithm used was the Extreme Gradient Boosting (XGBoost). Logarithmic loss and binary classification error rate were selected to monitor step by step the algorithm performance and stop it when the improvement subsides. Parameters of the models were tuned through a tenfold CV.

##### Model performance

Performance evaluation was made for the three different datasets, i.e., training set (with tenfold CV), test set, and hold-out set, and for each of them, accuracy (ACC), precision, sensitivity (SENS), and F1-score were calculated (ACC over 80% in all sets, PRC: 80%, 71%, 63%; SE: 85%, 86%, 77%; F1-score: 82 ± 5%, 78%, and 70%, respectively). From these results, there are more false positives than false negatives, indicating the maximization of the identification of very bitter compounds.

From the analysis of the feature importance, the algorithm pointed out the role of the molecule’s size and molar refractivity (a measure of polarizability) in determining the bitterness level, suggesting also a correlation between molecule size and bitter intensity.

BitterIntense applied to toxic databases, i.e., FocTox and CombiTox datasets, revealed that only a small portion (about 10%) of toxic substances are intensively bitter. The use of the BitterIntense model to the DILIrank dataset allows the evaluation of the correlation between bitterness level and hepatotoxicity, showing that most of the drugs (729) were classified as NVB. Then, approved and experimental compounds from Drugbank database (10,170 compounds) and natural compounds from NPatlas (24,805 compounds) were screened, showing that almost half of microbial natural products, but only 23.7% of drug candidates are predicted as VB. Finally, 34 potential drug candidates against COVID-19 retrieved from “Coronavirus Information–IUPHAR/BPS Guide to Pharmacology” were classified, showing that 41.2% of them are likely VB, thus significantly higher than the percentage of VB drug compounds from Drugbank, suggesting a possible involvement of the bitter taste and bitter receptors in this disease.

### Umami prediction

#### iUmami-SCM

iUmami-SCM is the first umami taste predictor based on umami peptide primary sequence information [28]. iUmami-SCM is a webserver (http://camt.pythonanywhere.com/iUmami-SCM) and related datasets are available on GitHub (https://github.com/Shoombuatong/Dataset-Code/tree/master/iUmami).

##### Data preparation and model construction

The dataset, known as UMP442, contains 140 proved umami peptides and 304 bitter structures taken from iBitter-SCM as negative samples (see also *Taste and food-related databases* for further details). Interestingly, the peptide length of both positive and negative samples is less than 10 amino acid residues. UMP442 was divided randomly into two parts keeping the unbalancing between the positive and negative data: the training set, made up of 80% of the dataset, was employed for the generation of an initial scoring card with a statistical approach and its optimization through a GA algorithm and the independent set (UMP-IND), composed of 20% of the dataset, was employed for performance evaluation. Dipeptides propensity scores and informative physicochemical properties were employed as features in this model.

##### Model performance

Prediction performance depends on the optimal dipeptide propensity score (opti-DPS), and therefore, 10 opti-DPS were evaluated with a 10-fold CV and compared with the initial dipeptide propensity score (init-DPS). Notably, compared to other traditional ML methods (DT, KNN, MLP, NB, SVM, and RF), iUmami-SCM demonstrated better performance.

iUmami-SCM reached on the test set accuracy of 86%, MCC of 68%, AUC of 89.8%, sensitivity of 71.4%, and a specificity of 93.4%. All these reported performances were calculated on the opti-DPS. However, due to the reduced numbers of peptides used for the model construction, iUmami-SCM presents as a major shortcoming a limited ability to correctly generalize the prediction.

### Multi-taste prediction

#### BitterSweet forest

BitterSweetForest is an open-access model based on KNIME created in 2018. This machine learning classifier predicts the sweetness and the bitterness of chemical compounds using binary fingerprints [58].

##### Data preparation and model construction

The dataset contains 517 artificial and natural sweet compounds, derived from SuperSweet, and 685 bitter molecules, taken from BitterDB. Instant Jchem software was employed for molecules standardization. All duplicated molecules were removed. Four different binary fingerprints were calculated with RDKit node in KNIME: Morgan fingerprint, Atom pair fingerprints, Torsion fingerprint, and Morgan Feat fingerprints. Training and test sets were obtained with an 80:20 partitioning scheme, keeping the balance between the two classes. To avoid overfitting, a leave-one-out cross-validation (LOO) was performed. A Random Forest with Tree Ensemble Learner and Predictor nodes in KNIME [80] was implemented, and a Bayesian-based features detection was applied to analyze the important and frequent features.

##### Model performance

The model was evaluated with several performances: accuracy, sensitivity, specificity, precision, *F*-score, ROC-AUC, and Cohen’s kappa. The BitterSweet model reached accuracy of 96.7%, AUC of 98%, and sensitivity of 91% and 97% for sweet and bitter prediction, respectively. Bayesian-based feature detection emphasized the independence between the top ten features of sweet and bitter molecules, despite the two molecule sets appearing to show similar characteristics.

The performance was also calculated in an external validation set, which includes bitter, sweet, and tasteless molecules. Despite tasteless molecules are not included in the training dataset, the model provided good results, and this suggests the features employed are specific for bitter and sweet prediction. Interestingly, the screening of SuperNatural II, DrugBank approved drug molecules and ProTox, including oral toxicity compounds, showed that most molecules exhibited bitter features and toxic substances are normally bitter.

#### BitterSweet

BitterSweet is a freely accessible tool created in 2019 to classify bitter-sweet molecules [59]. To boost the progress in the knowledge of bitter-sweet taste molecular basis, the creators of this tool make all datasets, models, and even end-to-end software publicly available (https://cosylab.iiitd.edu.in/bittersweet/; https://github.com/cosylabiiit/bittersweet).

##### Data preparation and model construction

The dataset was created avoiding two problems observed in previous studies: the use of *unverified flavor molecules* in the training dataset, as happened in BitterPredict and BitterX, and the use of *only experimentally verified data*, leading to a drastic reduction in the dataset size. The dataset contains around thousands of chemicals among bitter, non-bitter, sweet, and non-sweet compounds retrieved from literature [27, 29, 53], pre-existing databases, i.e., SuperSweet, The Good Scents Company Database, BitterDB and books, i.e., Fenaroli’s Handbook of Flavor Ingredient And Biochemical Targets of Plant Bioactive Compounds. Moreover, as the control for bitter and sweet prediction, tasteless, and contrasting taste compounds, derived from ToxNet, TastesDB, and Fenaroli’s Handbook of Flavor Ingredient, were introduced in the dataset. The canonical SMILES were extracted through OpenBabel [81]. Duplicate structures, peptides, molecules with only three atoms, and salt ions were removed, while only the lowest energy conformer for each molecule was retained. The chirality of the molecule was preserved. The 3D conformation and protonation state at physiological pH (7 ± 0.5) were carried out using Epik [82] and LigPrep (Schrödinger).

The training dataset for bitter/non-bitter prediction included 813 bitter molecules as positive data and 1444 sweet and tasteless molecules as the negative set, while for sweet/non-sweet prediction, it consisted of 1139 sweet molecules as the positive set and 1066 bitter and tasteless compounds as the negative set. The test dataset was formed by 105 bitter and 66 non-bitter structures in the bitter prediction and 108 sweet and 53 bitter/tasteless molecules in sweet prediction. Moreover, a fivefold stratified CV was performed to assess the model parameters.

Five sets of molecular descriptors, both commercial and open-source, were employed to create an exhaustive set of features: Physicochemical and ADMET descriptors from Canvas, Extended-Connectivity Fingerprints (ECFP), 2D Molecular Descriptors and 2D/3D Molecular Descriptors from Dragon 2D and Dragon 2D/3D and 2D Topological and Structural Features from ChemoPy. Due to the high number of molecular descriptors, the Boruta algorithm [83] was employed to remove irrelevant features and principal component analysis (PCA) to get the maximum variance. Three different ML-based models were employed, i.e., Random Forest (RF), Ridge Logistic Regression (RLR) and Adaboost (AB). For each algorithm and each prediction, the five set of molecular descriptors were evaluated separately.

##### Model performance

BitterSweet model performance was evaluated employing several metrics, including the Area Under the Precision-Recall Curve (PR-AUC), ROC-AUC, F1-score, sensitivity, and specificity. The models that best discriminate the sweet non-sweet dichotomy were AB and RF trained after the Boruta algorithm, while PCA performed better than the Boruta algorithm only when coupled with RLR. In contrast, the algorithm that best predicts bitter taste was RLR, while the RF performed well across all molecular descriptor sets. Furthermore, PCA would seem to perform better than the Boruta algorithm. The best descriptors for the sweet prediction were Dragon 2D features, whereas the open-source ChemoPy performed better for bitter prediction.

In conclusion, the best BitterSweet model (with AB algorithm after the Boruta feature selection and Dragon 2D/3D features) achieved these performances: ROC-AUC of 88.3%, PR-AUC of 95%, the sensitivity of 79%, the specificity of 88%, and the F1-score of 86%. However, in their online tool, they employed ChemoPy descriptors with the RF-PCA algorithm as the performance of open-source descriptors were comparable to those obtained through proprietary software. The results achieved with the BitterSweet model: ROC-AUC of 84% and 88%, PR-AUC of 93% and 93%, the sensitivity of 59% and 79%, the specificity of 94% and 85%, and the F1-score of 73% and 84%, and all the results were reported for sweet and bitter prediction, respectively.

The BitterSweet model was also applied to several specialized chemical databases, i.e., SuperSweet, FlavorDB, FooDB, DSSTox, SuperNatural II, and DrugBank, revealing that the majority of natural, toxic, and drug-like molecules are bitter, whereas for food molecules, there was the same amount of bitter and sweet molecules.

In conclusion, despite the high accuracy of the BitterSweet open-source predictors, its utility is limited to individual compounds, and not for different compounds when present in a mixture.

#### Virtual taste

VirtualTaste platform is the first freely available web server able to predict three taste qualities (sweet, bitter, and sour), thanks to three dedicated tools, i.e., VirtualSweet, VirtualBitter, and VirtualSour, respectively (http://virtualtaste.charite.de/VirtualTaste/) [60]. The input of the web-based platform is the two-dimensional structure of the chemical compound, and the output is the prediction of the chemical’s taste profile and the targeted TAS2R receptors in case of a bitter prediction.

##### Data preparation and model construction

The dataset contains 2011 sweet compounds, collected from the SuperSweet database and BitterSweetForest tool, 1612 bitter molecules derived from the BitterDB database and BitterSweetForest tool, and 1347 sour compounds obtained from ChEMBL [35] and manually edited from the literature sources [84]. Furthermore, the bitter receptor data contain 356 ligands that interact with TAS2Rs receptor extracted from BitterDB, ChEMBL and literature. Different structures were removed from the database, such as ambiguous compounds, salt and inconclusive entries, and then standardized through RDkit in KNIME [80]. Each dataset was split into two parts, preserving the positive/negative ratio: the training set made up of 80% of each set of molecules, i.e., 1068 molecules for sweet, 1289 compounds for bitter and 1214 structures for sour, and the remaining chemicals were employed for the external validation set. The inactive dataset used in each model were different: bitter and tasteless compounds were used as inactive compounds for the sweet prediction and sweet and tasteless compounds were employed as inactive compounds for the bitter prediction, while the sour prediction used a ligand-based approach due to the pH and acid influence present in foods. Moreover, a tenfold CV was applied for model optimisation, keeping the ratio of active and inactive structures constant.

Each VirtualTaste model was based on an RF algorithm, following BitterSweetForest, the previous tool developed by the same research group [58]. To deal with the negative effect of the unbalanced dataset, different data sampling methods were applied: the Synthetic Minority Over-Sampling Technique-using Tanimoto Coefficient (SMOTETC) technique for VirtualSweet, the Synthetic Minority Over-Sampling Technique-using Value Difference Metric (SMOTEVDM) method for VirtualBitter and the Augmented Random Over-Sampling (AugRandOS) method for VirtualSour [85]. A similarity-based method was employed for the prediction of bitter receptors [86]: the similarity between the query molecule and known bitter compounds is evaluated using the Tanimoto Coefficient and the relative target bitter receptor is then consequently predicted.

VirtualSweet and VirtualBitter models were also used for taste prediction of approved drugs and natural compounds—1969 chemical compounds from DrugBank database and 326,000 from SuperNatural II.

##### Model performance

Five performance metrics both in the 10-fold CV and in the external evaluation set were utilized. VirtualSweet reached on the external validation 95% for ROC-AUC, 89% for the accuracy, 92% for specificity, 86% for sensitivity, and 88% for F1-score; VirtualBitter 96% for ROC-AUC, 90% for the accuracy, 97% for specificity, 88% for sensitivity, and 88% for F1-score; VirtualSour 99% for ROC-AUC, 97% for the accuracy, 99% for specificity, 80% for sensitivity, and 84% for F1-score. In conclusion, VirtualTaste is the first tool able to predict with reliable results three different taste qualities and achieve comparable or better performance compared to similar tools.

## Discussion

In this section, a detailed comparison between all the above-described taste prediction tools is provided. The performance of the classification and regressor models is summarized in Tables 4 and 5, respectively. It is noteworthy that these comparative results were not obtained on the same datasets and different evaluation metrics were used in each analyzed work. Data in the tables refer to performance on the test set.

**Table 4:** Performance on the test set of the taste prediction classification tools

Taste	Tool	Performance (%)							
		AUC	SE	SP	ACC	PRC	NER	F1	MCC
Sweet	Rojas Sweet Predictor	/	88	82	/	/	85	/	/
	e-Sweet	/	86	94	91	90	90	88	81
Bitter	BitterX	95	92	91	92	91	/	/	/
	BitterPredict	/	77	86	/	/	/	/	/
	e-Bitter	/	98	81	92	/	/	94	82
	iBitter-SCM	90	84	84	84	/	/	/	69
	BERT4Bitter	96	94	91	92	/	/	/	84
	iBitter-Fuse	93	94	92	93	/	/	/	86
	BitterIntense	/	86	81	83	71	/	78	/
Bitter–Sweet	BitterSweet Forest	98	91	97	97	/	94	92	/
	BitterSweet (Sweet)	84	59	94	/	/	77	73	/
	BitterSweet (Bitter)	88	79	85	/	/	82	84	/
Umami	iUmami-SCM	90	71	93	87	/	/	/	68
Sweet–Bitter–Sour	VirtualSweet	95	86	92	89	/	/	88	/
	VirtualBitter	96	88	97	90	/	/	88	/
	VirtualSour	99	80	99	97	/	/	84	/

**Table 5:** Performance on the test sets of the sweet prediction regression tools

Tool	Performance		
	*R*^2^	MAE	MSE
Chéron Sweet Regressor	0.85	/	/
Goel Sweet Regressor	0.83	0.39	0.23
Predisweet	0.74	0.50	0.44

At present, it is evident that there is a net prevalence of tools for predicting sweet and bitter tastes. It is worth noting that only one example to predict the umami taste (iUmami-SCM) and one for the sour taste (VirtualSour in VirtualTaste) exist, and no tools for predicting the saltiness have been released, as far as the authors know. Moreover, the definition of a regression algorithm was possible only for the sweet taste (Chéron Sweet Regressor, Goel Sweet Regressor, and PrediSweet) since, to date, no database for other taste sensations provides quantitative data concerning the level of the perceived taste. However, BitterIntense, despite being a classification algorithm, discriminates between “*very bitter*” and “*non very bitter*” compounds, thus accessing the level of bitterness of query molecules.

Despite all prediction tools employ a different methodology, a common structural features can be noticed among all of them, which is typical of most ML workflows: (i) the definition of a compound database also including the respective taste, preferably experimentally validated; (ii) the compound featurization, i.e., the derivation of effective molecular descriptors; (iii) the dataset splitting into training and test sets (and in some cases also a validation set); (iv) the choice of the ML method for the classification/regression; (v) performance evaluation and validation. It is worth mentioning that most of the discussed algorithms followed the guidelines defined by the Organization for Economic Co-operation and Development (OECD), which indicates the strategies for correct development and validation of robust QSAR models: (i) a defined endpoint; (ii) an unambiguous algorithm; (iii) a defined domain of applicability; (iv) appropriate measures of goodness-of-fit, robustness and predictivity; (v) a mechanistic interpretation, if possible [87].

Several tools and methods, both proprietary and open-source, were used to derive molecular descriptors, including Dragon, Canvas (Schrödinger), Extended-connectivity Fingerprint (ECFP), RDKit, Mordred, and ChemoPy. It is important to note that open-source descriptors (RDKit, Mordred, ChemoPy) have been shown not to remarkably affect the performance of the PrediSweet model and to reach similar results if compared to results obtained with Dragon descriptors [50]. Similarly, in BitterSweet the best descriptors for the sweet prediction were Dragon 2D features, but the open-source ChemoPy performed better for bitter prediction [59]. This represents a very important achievement in making these tools available to a wide audience and in broadening the horizons of research in this field. Furthermore, 2D molecular descriptors are less time-consuming to be computed and less subject to variations caused by slightly different molecules 3D conformations. On the other hand, 3D descriptors can also account for specific molecule conformations, such as different conformers/isomers, and spatial properties. Therefore, the possibility to obtain very good results also using 2D descriptors allows designing faster tools suitable for screening very large databases.

Several algorithms have been applied for taste prediction, including RF, SVM, SVR, QSTR, GFA, ANN, KNN, GBM, DNN, AB, SCM, and XGBoost. Multiple Linear Regression (MLR) and Support Vector Machine (SVM) are among the first models for binary classification. These models were exceeded by tree-based models, i.e., Random Forest (RF) or AdaBoost (AB), and Neural Network (NN), which support multiclass classification and work very well in the non-linear range if they have a sufficiently large number of database elements. Generally, NN and SVM perform better with continuous and multidimensional features, but they need a large sample size to increase their prediction accuracy [88]. Even though NNs, and in particular ANNs and DNNs, are being widely employed in taste prediction, they are characterized by difficulties in optimizing parameters, a high computational cost, and are less explainable. Moreover, probabilistic methods, i.e., Naive Bayes, are not widely used in taste prediction. These methods work well with less training data, but would be better employed when the features are mutually independent.

To enhance model performance and to increase the understanding of the model, a feature selection was normally applied, such as the V-WSP unsupervised variable reduction method and genetic algorithm-based technique [29, 51, 56], feature importance obtained from the RF [49, 54], the Boruta algorithm, and the PCA [59]. However, none of these approaches consider the multi-objective nature of the dimensionality reduction techniques and thus fail to balance between the objectives of optimizing prediction performance measured in multiple classifications and regression metrics, minimizing the number of selected features and maximizing the overall interpretability/explainability of the derived prediction models. Moreover, not only feature selection but also normalization/standardization can further improve model performance, such as in the case of the *flatkinson* standardization method [50], as well as modern techniques to handle the class imbalances in the data such as SMOTE [89]. Finally, all existing methods lacked a strict definition of negative datasets with most of them using random compounds as negative datasets and thus jeopardizing the prediction performance and generalization properties of the models.

As also defined by the OECD guidelines, another relevant aspect in the development of the prediction tool is the definition of the *applicability domain* (AD), which indicates the reliability of the prediction evaluating if investigated compounds are within the chemical space of the training data. In this context, PrediSweet, along with an applicability domain, developed a *reliability domain*, which considers the density of information around the compound, and a *decidability domain*, which evaluates the confidence of the prediction. The PrediSweet chemical space of the dataset used (SweetenersDB) was compared with the most comprehensive sweet database (SuperSweet): more than 99.5% of the compounds in SuperSweet are structurally similar to a representative structure in the SweetenersDB, suggesting the large sweeteners spectrum covered by the SweetenersDB. e-Bitter and e-Sweet used the ECFP-based Tanimoto similarity between the query and the five closest neighboring molecules in the training set. Similarly, the Rojas Sweet Predictor developed an AD using a threshold on the Jaccard–Tanimoto average distance between the query molecule and the compounds in the dataset. BitterPredict AD, known as *Bitter Domain*, includes molecules with molecular weight MW ≤ 700 and hydrophobicity − 3 ≤ AlogP ≤ 7: all used datasets were previously filtered using this domain to ensure the reliability of the prediction. In BitterSweet, a query molecule is considered inside the applicability domain, if its median Euclidean distance from similar compounds in the training set is below a selected threshold. Interestingly, BitterSweet covered a remarkably wider applicability domain than the Rojas Sweet Predictor while achieving similar performance.

One of the main advantages of the reported prediction tools is their ability to fast screening huge databases of compounds and to estimate the number of compounds associated with a specific taste. A granular and detailed screening was performed by BitterPredict on DrugBank approved (1375 compounds), FooDB (13,588 compounds), Natural Products Dataset from ZINC15 (27,474 compounds), and ChEBI (27,015 compounds) datasets, showing that the percentages of bitter molecules within the Bitter Domain found in these databases are 65.94%, 38.36%, 77.21%, and 43.71%, respectively. Moreover, since bitter taste is associated with toxic compounds or compliance problems, the same authors used their subsequent tool, namely BitterIntense, to screen toxic databases, i.e., FocTox, CombiTox datasets, and DILIrank, experimental compounds from DrugBank database (10,170 compounds), natural compounds from NPatlas (24,805 compounds) and 34 potential drug candidates against COVID-19 retrieved from “Coronavirus Information–IUPHAR/BPS Guide to Pharmacology”. Interestingly, only a small portion of toxic compounds are intensively bitter, but 41.2% of COVID-19 candidate drugs were predicted as very bitter (VB). Moreover, BitterSweet was applied to several specialized chemical databases (SuperSweet, FlavorDB, FooDB, DSSTox, SuperNatural II, and DrugBank), revealing that most natural, toxic, and drug-like chemicals are bitter, whereas the same amount of bitter and sweet molecules are present in foods. BitterSweet Forest was applied on SuperNatural II, DrugBank approved drug molecules and ProTox, and showed that toxic substances are typically bitter. In line with the previous results, VirtualSweet and VirtualBitter models were applied on approved drugs from DrugBank and natural compounds from SuperNatural II: notably, most of the approved drugs and most of the natural compounds were predicted as bitter, and only a small portion of these databases was classified as sweet.

## Conclusions

The present review aims at summarizing the main scientific advances in the field of taste prediction supported by ML-based algorithms. We discussed the main available database containing food-related compounds and molecules with known taste, the main tools employed to predict the taste.

From the analysis of the databases, we pointed out two specific databases for the sweet taste, i.e., SuperSweet, which is the most comprehensive DB for sweeteners, and SweetenersDB, which collects 316 sweeteners with a relative value of sweetness. For the bitter taste, BitterDB represents the most granular and complete database, with a very intuitive and user-friendly web server that allows the download of more than a thousand bitter compounds. BitterDB, as well as BitterPredict and BitterIntense, was developed by the Niv Lab (https://biochem-food-nutrition.agri.huji.ac.il/mashaniv), which has provided incredible advances in the comprehension of the bitter taste in recent years. A lot of effort has been made to develop methods specifically for the prediction of bitter peptides and another research group has continuously improved its tools publishing three consecutive works, namely iBitter-SCM, BERT4Bitter, and iBitter-Fuse, in the last few years. These tools are paramount for the fast and reliable classification of huge databases of bitter peptides and for their rational de novo design, especially considering the emerging role of this class of compounds in the drug and nutritional research field. Furthermore, the UMP442 database developed during the implementation of iUmami-SCM is probably the most complete and ready-to-use database of umami and non-umami molecules, since it is available from GitHub. In this context, the Umami Database seems a very promising source of information, but the availability of data is limited, it is impossible to obtain data from the webserver and no umami prediction tool which uses this source has been found in the previous literature. It would be incredibly valuable to have access to the resources of such a database in the future. Finally, as far as the authors know, no publicly available databases for sour and salty tastes are available and the only attempt to generate a sour dataset was made in the development of VirtualTaste. However, the used sour dataset has not been made public. To date, the multiplicity and diversity of sources make it very complex to obtain a unified DB collecting a huge amount of compounds for each taste sensation. The authors insist also on the need for developing complete databases that include all the relevant information for each entry (SMILES, InChI, IUPAC nomenclature, etc.) to avoid any possible error in compound processing. Moreover, the definition of exhaustive databases would be essential for a correct definition of the molecular descriptors to be employed, due to the great number and variety of both open-source and proprietary descriptors.

Similarly to the taste databases, prediction tools for sweet and bitter have been more developed during the last years. Among several examples of sweet and bitter classification tools, some proposed methods can even predict the level of sweetness (Chéron Sweet Regressor, Goel Sweet Regressor, and PrediSweet) and BitterIntense can discriminate between “very bitter” and “non very bitter compounds”. Only a few reported tools were able to discriminate more than one taste sensation. BitterSweet and BitterSweet Forest are interesting examples of tools able to consider the dichotomy of sweet and bitter tastes [59]. These tools can be pivotal for the detection of natural and synthetic compounds with a pleasant taste and without adverse effects. Furthermore, VirtualTaste is the only available tool able to predict three taste sensations (sweet, bitter, and sour) and the only one able to predict the sour taste (VirtualSour).

Among the 16 reported taste prediction tools, BitterX, BitterSweet, PrediSweet, iBitter-SCM, BERT4Bitter, iBitter-Fuse, iUmami-SCM, and VirtualTaste provide web server applications, which allow the taste prediction using the SMILE/Fasta format, directly drawing the molecule or by uploading a file. On the other hand, e-Sweet, e-Bitter, and BitterPredict only provide freely accessible code available from Dropbox or GitHub. From the authors’ point of view, the development of a web interface represents a considerable strength, since it allows the tool to be used even by people who are not experts in the use of these applications. Finally, BitterSweetForest, Rojas Sweet Predictor, Goel Sweet Regressor, Chéron Sweet Regressor, and BitterIntense do not have any web server or code publicly available.

It is worth mentioning that in addition to the five basic tastes, other taste qualities may be important and related to specific food ingredients. In this context, some recent publications suggested the fat taste as another basic taste quality [3, 4, 90]. Interestingly, fatty acid detection seems to decrease as a consequence of a fat-rich diet and with a great impact on obesity disease [91]. Therefore, the prediction of fat taste would represent a groundbreaking objective for future tools, considering the impact of fat intake on human health status.

Furthermore, the use of methods capable of predicting the molecular interactions of tastants and relative taste receptors could lead to significant improvements in the predictive capabilities of these tools and to great strides in understanding the physicochemical characteristics and mechanisms underlying taste perception.

## Supplementary Information

Below is the link to the electronic supplementary material.

Supplementary file1 (DOCX 48 kb)

## Abbreviations

R: Correlation coefficient
SD: Standard deviation
SP: Specificity
SE: Sensitivity
ACC: Accuracy
MCC: Matthews’s coefficient correlation
PRC: Precision
NER: Non-error rate
CV: Cross-validation
ROC-AUC: Area under a receiver-operating characteristic curve
VB: Very bitter
NVB: Not very bitter
Init-DPS: Initial dipeptide propensity score
Opti-DPS: Optimal dipeptide propensity score
RF: Random forest
SVM: Support vector machine
SVR: Support vector regression
KNN: K- nearest neighbors
GBM: Gradient boosting machine
DNN: Deep neuron network
ANN: Artificial neural network
GFA: Genetic function approximation
ECFP: Extended-connectivity fingerprint
PCA: Principal component analysis
OECD: Organization for economic co-operation and development
AD: Applicability domain
CM: Consensus models
