# Supplementary material for: A survey on computational taste predictors
Source: Eur Food Res Technol. 2022 May 26;248(9):2215–35. doi: 10.1007/s00217-022-04044-5 (PMC9134981; doi:10.1007/s00217-022-04044-5)
Supplement: Supplementary file 1 — Supplementary file1 (DOCX 48 kb) [file 217_2022_4044_MOESM1_ESM.docx]

# Supplementary Materials:

***Area under the curve (AUC)*** measures the two-dimensional area under the ROC curve. It provides an aggregate measure of performance by examining all possible classification thresholds. The AUC is scale-invariant and is an indicator of how well predictions are classified, rather than their absolute values. The value is included between 0 and 1, and it is expressed in percentage. The more the value is far from 1, the more the model predicts wrong. An area of 0.5 corresponds to a random classifier.

***Sensitivity (SE)*** represents the true positive rate, the number of positive data correctly classified.

$$Sensitivity= \frac{True Positive}{True Positive+False Negative}$$

***Specificity (SP)*** represents the true negative rate, the number of negative data correctly classified.

$$Specificity= \frac{True Negative}{True Negative+False Positive}$$

***Accuracy (ACC)*** is the ratio of the number of correct predictions to the total number of input samples, it represents how well the ML algorithms correctly classify the samples.

$$Accuracy= \frac{True Positive+ True Negative}{True Positive+False Positive+ True Negative+False Negative}$$

***Precision (PRC),*** or ***Positive Predictive Value (PPV),*** represents the is the number of the real positive samples divided by the number of positive results predicted by the classifier.

$$Precision= \frac{True Positive}{True Positive+False Positive}$$

***Non-Error Rate (NER)*** represents the arithmetic mean of Sensitivity and Specificity in binary classification.

$$Non-Error Rate= \frac{Sensitivity+ Specificity}{2}$$

***F_β_-score*** allows weighting precision and recall, especially in an unbalanced dataset.

$$F_{\beta}-score =(1+ \beta^{2}) \frac{Precision*Recall}{\beta^{2}*Precision+Recall}$$

For β = 1, F_1_-score (F1) is the Harmonic Mean between precision and recall. It tells how precise and robust is your classifier. High precision but lower recall means an extremely accurate classifier, but it misses a large number of instances difficult to classify. The range of the F_1_-score is [0,1] and the greater it is, the better is the model performance.

***Matthew's correlation coefficient (MCC)*** is a single-value metric that summarizes the confusion matrix. This coefficient has a high value only if it classifies correctly both positive and negative elements. When the classification is perfect, MCC value is 1.

$$MCC =\frac{TP*TN - FP*FN}{\sqrt{\left[ \left( TP + FP \right)*\left( TP + FN \right)*\left( TN + FP \right)*\left( TN + FN \right) \right]}}$$

***Table S1.*** *Summary of the main recent taste prediction tools, including the methods, the datasets and the molecular descriptors employed.*

| **Reference** | **Method** | **Dataset** | | | **Molecular Descriptors** |
| --- | --- | --- | --- | --- | --- |
|  |  | **Taste** | **Source** | **#** |  |
| **Chéron Sweet Regressor** [24] | Sweet Regressor (RF, SVR) | **Sweet** | SweetenersDB | 316 | Dragon |
| **Rojas Sweet Predictor** [29] | Sweet Classifier (QSTR) | **Sweet** | Sweet | 435 | 2D molecular descriptors (Extended-connectivity Fingerprint (ECFP), Dragon) |
|  |  | **Non-Sweet** | Bitter | 81 |  |
|  |  |  | Tasteless | 133 |  |
| **Goel Sweet Regressor** [48] | Sweet Regressor (GFA, ANN) | **Sweet** | Literature [63–67] | 487 | Material Studio |
|  |  |  |  |  |  |
| **e-Sweet** [49]  **[**https://bit.ly/3wFy4ER**]** | Sweet Classifier (KNN, SVM, GBM, RF, DNN) | **Sweet** | SuperSweet | 530 | Extended-connectivity Fingerprint (ECFP) |
|  |  |  | SweetenersDB |  |  |
|  |  |  | TasteDB |  |  |
|  |  |  | BitterSweet Forest |  |  |
|  |  | **Non-Sweet** | BitterDB | 718 |  |
|  |  |  | Tasteless (TastesDB) | 132 |  |
| **Predisweet** [50]  **[**https://bit.ly/3reop7a**]** | Sweet Regressor (AB) | **Sweet** | SweetenersDB | 316 | Dragon and open-source (RDKit, Mordred, ChemoPy) |
| **BitterX** [51]  **[**https://bit.ly/3wJYa9O**]** | Bitter Classifier  (SVM) | **Bitter** | BitterDB | 539 | Descriptors from the Handbook of Molecular Descriptors (Todeschini and Consonni, 2007) |
|  |  | **Non-Bitter** | In-house experiments | 20 |  |
|  |  |  | Available Chemicals Directory (ACD) | 519 |  |
| **BitterPredict** [53]  **[**https://bit.ly/3igrzmQ**]** | Bitter Classifier (AB) | **Bitter** | BitterDB | 632 | Canvas (Schrödinger) |
|  |  |  | TastesDB | 59 |  |
|  |  | **Non-Bitter** | Fenaroli’s Handbook of Flavor ingredients | 1451 |  |
|  |  |  | Non-bitter (literature) | 35 |  |
|  |  |  | Sweet [29] | 336 |  |
|  |  |  | Tasteless [29] | 130 |  |
| **e-Bitter** [54]  **[**https://bit.ly/3epWzQq**]** | Bitter Classifier (KNN, SVM, RF, GBM, DNN) | **Bitter** | BitterDB | 707 | Extended-connectivity Fingerprint (ECFP) |
|  |  |  | TastesDB |  |  |
|  |  |  | Rodgers et al., 2006 |  |  |
|  |  | **Non-Bitter** | Tasteless (TasteDB) | 132 |  |
|  |  |  | Non-bitter (BitterX) | 17 |  |
|  |  |  | Sweet (SweetenersDB; SuperSweet; [29, 72]) | 443 |  |
| **iBitter-SCM** [26]  **[**https://bit.ly/2VGyXAg**]** | Bitter Peptides Classifier (SCM) | **Bitter** | Literature (peptides w/ experimental bitterness) | 320 | Dipeptide composition (DPC) |
|  |  | **Non-Bitter** | Randomly from BIOPEP | 320 |  |
| **BERT4Bitter** [55] [<https://bit.ly/2WecTxf>] | Bitter Peptides Classifier (BERT) | **Bitter** | Literature (peptides w/ experimental bitterness) | 320 | Dipeptide composition (DPC) |
|  |  | **Non-Bitter** | Randomly from BIOPEP | 320 |  |
| **iBitter-Fuse** [56]  [<https://bit.ly/3BmC547>] | Bitter Peptides Classifier (SVM) | **Bitter** | Literature (peptides w/ experimental bitterness) | 320 | DPC, AAC, PAAC, APAAC, AAI |
|  |  | **Non-Bitter** | Randomly from BIOPEP | 320 |  |
| **BitterIntense** [57] | Bitter Intensity Classifier (XGBoost) | **VB** | BATA model | 246 | Canvas (Schrödinger) |
|  |  |  | BitterDB |  |  |
|  |  |  | AnalytiCon’s repository |  |  |
|  |  | **NVB** | Random from non-bitter of BitterPredict | 404 |  |
|  |  |  | BitterDB |  |  |
|  |  |  | AnalytiCon’s repository |  |  |
| **BitterSweetForest** [58] | Bitter/Sweet Classifier (RF) | **Sweet** | SuperSweet | 517 | RDKit (Binary fingerprints) |
|  |  | **Bitter** | BitterDB | 685 |  |
| **BitterSweet** [59]  **[**https://bit.ly/3rd7Att**]** | Bitter/Sweet Classifier  (AB, RF) | **Bitter** | TasteDB | 918 | Canvas (Physicochemical and ADMET) Dragon 7 (Extended Connectivity Fingerprints, 2D Molecular Descriptors and 3D Molecular Descriptors) ChemoPy (2D Topological and Structural Features) |
|  |  |  | Rodgers et al., 2006 |  |  |
|  |  |  | Fenaroli's Handbook of Flavor Ingredient |  |  |
|  |  |  | Biochemical Targets of Plant Bioactive Compounds |  |  |
|  |  |  | BitterDB |  |  |
|  |  |  | The Good Scents Company Database |  |  |
|  |  |  | BitterPredict (Phyto-Dict., Bitter-New, UNIMI) |  |  |
|  |  | **Non-Bitter** | Bitter Predict (Phyto-Dict., UNIMI) | 1510 |  |
|  |  | **Sweet** | TastesDB | 1205 |  |
|  |  |  | Fenaroli's Handbook of Flavor Ingredient |  |  |
|  |  |  | Biochemical Targets of Plant Bioactive Compounds |  |  |
|  |  |  | SuperSweet |  |  |
|  |  |  | The Good Scents Company Database |  |  |
|  |  | **Non-Sweet** | Tasteless (TasteDB, Fenaroli' s Handbook, ToxNet) | 1171 |  |
|  |  |  | Bitter molecules |  |  |

| **iUmami-SCM** [28]  **[**https://bit.ly/3hJs9uf**]** | Umami Classifier (SCM) | **Umami** | BIOPEP-UWM | 140 | Dipeptide composition (DPC) |
| --- | --- | --- | --- | --- | --- |
|  |  |  | Literature [41, 43, 46, 92–94] |  |  |
|  |  | **Non-Umami** | Bitter: iBitter-SCM | 304 |  |
| **VirtualTaste** [60]  **[**https://bit.ly/2UfVFPi**]** | Multi-taste classifier (RF) | **Sweet** | SuperSweet | 2011 | *not reported* |
|  |  |  | BitterSweetForest |  |  |
|  |  | **Bitter** | BitterDB | 1612 |  |
|  |  |  | BitterSweet Forest |  |  |
|  |  | **Sour** | Manually edited from ChEMBL | 1347 |  |
